# Bariatric surgery: trends in utilization, complications, conversions and revisions

**DOI:** 10.1007/s00464-024-10985-7

**Published:** 2024-06-20

**Authors:** Justin L. Hsu, Sherin Ismail, Maggie M. Hodges, Chris B. Agala, Timothy M. Farrell

**Affiliations:** 1 Department of Surgery, University of Toledo College of Medicine and Life Sciences, Mail Stop 1095, 3000 Arlington Ave, Toledo, OH 43614 USA; 2 Department of Epidemiology, Gillings School of Global Public Health, University of North Carolina at Chapel Hill, Chapel Hill, USA; 3 Department of Surgery, School of Medicine, University of North Carolina at Chapel Hill, Chapel Hill, USA

**Keywords:** Bariatric surgery, Sleeve gastrectomy, Gastric bypass, Duodenal switch, Weight loss, Morbid obesity

## Abstract

**Background:**

Sleeve gastrectomy (SG) increased in popularity after 2010 but recent data suggest it has concerning rates of gastroesophageal reflux and need for conversions. This study aims to evaluate recent trends in the utilization of bariatric procedures, associated complications, and conversions using an administrative claims database in the United States.

**Methods:**

We included adults who had bariatric procedures from 2000 to 2020 with continuous enrollment for at least 6 months in the MarketScan Commercial Claims and Encounters database. Index bariatric procedures and subsequent revisions or conversions were identified using CPT codes. Baseline comorbidities and postoperative complications were identified with ICD-9-CM and ICD-10 codes. Cumulative incidences of complications were estimated at 30-days, 6-months, and 1-year and compared with stabilized inverse probability of treatment weighted Kaplan–Meier analysis.

**Results:**

We identified 349,411 bariatric procedures and 5521 conversions or revisions. The sampled SG volume appeared to begin declining in 2018 while Roux-en-Y gastric bypass (RYGB) remained steady. Compared to RYGB, SG was associated with lower 1-year incidence [aHR, (95% CIs)] for 30-days readmission [0.65, (0.64–0.68)], dehydration [0.75, (0.73–0.78)], nausea or vomiting [0.70, (0.69–0.72)], dysphagia [0.55, (0.53–0.57)], and gastrointestinal hemorrhage [0.43, (0.40–0.46)]. Compared to RYGB, SG was associated with higher 1-year incidence [aHR, (95% CIs)] of esophagogastroduodenoscopy [1.13, (1.11–1.15)], heartburn [1.38, (1.28–1.49)], gastritis [4.28, (4.14–4.44)], portal vein thrombosis [3.93, (2.82–5.48)], and hernias of all types [1.36, (1.34–1.39)]. There were more conversions from SG to RYGB than re-sleeving procedures. SG had a significantly lower 1-year incidence of other non-revisional surgical interventions when compared to RYGB.

**Conclusions:**

The overall volume of bariatric procedures within the claims database appeared to be declining over the last 10 years. The decreasing proportion of SG and the increasing proportion of RYGB suggest the specific complications of SG may be driving this trend. Clearly, RYGB should remain an important tool in the bariatric surgeon’s armamentarium.

**Supplementary Information:**

The online version contains supplementary material available at 10.1007/s00464-024-10985-7.

Obesity is an epidemic disease that is rapidly growing, with a recent US national survey in 2018 showing the prevalence of obesity has risen from 30.5% in 1999 to 42.4% in 2018. [1] The prevalence of severe obesity has also almost doubled within that time period, from 4.7 to 9.2% [1]. Obesity and its associated medical comorbidities are estimated to have a medical cost of $170 billion annually with an economic burden of $1.72 trillion [2, 3]. Bariatric surgery remains the most effective and durable treatment option for obesity. Currently, laparoscopic sleeve gastrectomy (SG) is the most performed bariatric procedure, having surpassed laparoscopic Roux-en-Y gastric bypass (RYGB) in 2012 [4]. The overall volume of bariatric surgery increased by 60% from 2011 to 2018 with sleeve gastrectomy demonstrating a remarkable 451% growth trend [5]. However, the utilization of bariatric surgery in patients who could qualify remains low despite the significant improvement in perioperative risk reduction.

Revisional surgery had also been rising steadily since 2011, increasing more than three-fold through 2019 and representing the third most commonly performed bariatric procedure after SG and RYGB [6]. Despite the relative simplicity and low incidence of complications of SG, there has been evidence pointing at higher revision and conversion rates when compared to RYGB [7, 8]. Gastroesophageal reflux disease (GERD), insufficient weight loss, and weight regain are the most common reasons for revisions and conversions after SG. Studies have also shown that conversion from SG to RYGB carries a higher risk of serious complications when compared to primary RYGB [9]. Although the choice of primary procedure is typically dependent on patient factors and surgeon preference, it is important to recognize the risks for future revisions and to educate patients accordingly. This study aims to build on a previous retrospective review of nationwide commercial claims data by Chung et al., to evaluate recent trends in bariatric procedure utilization, and to expand upon the previous study by analyzing the associated incidence of complications, revisions, and conversions.

## Methods

The study was conducted using the IBM MarketScan® Commercial Claims and Encounters database. We included adult patients (18 years or older) who underwent bariatric procedures from 2000 to 2020 and had a continuous enrollment for at least 180 days before the index bariatric procedure. We identified the following bariatric procedures using CPT codes: CPT codes 43,644, 43,645 (laparoscopic RYGB), 43,846, 43,847 (open RYGB), 43,775 (laparoscopic SG), 43,770 (laparoscopic adjustable gastric banding (AGB)), 43,842 (vertical banded gastroplasty (VBG)), 43,845 (biliopancreatic diversion with duodenal switch (BPD/DS)). The baseline characteristics of the study cohort in the 6-months before the index procedure were identified using ICD-9-CM and ICD-10 codes. We used the first CPT code for a bariatric operation as the index procedure and recorded subsequent incidence of complications at 30-days, 6-months, and 1-year using ICD-9-CM, ICD-10, and CPT codes from administrative claims, where applicable. The full list of complications and codes used is shown in Appendix 1. Subsequent CPT codes after the index procedure were used to identify revisions and conversions. We also separately identified first and second revisions and/or conversions based on subsequent related CPT codes after the index procedure. Other non-revisional surgical interventions such as internal hernias, ulcer-related procedures, and unlisted procedures were recorded after 6-months and 1-year.

### Statistical analysis

The baseline characteristics were stratified by the type of procedure. Continuous variables were reported as either mean ± standard deviation (SD) if the data were normally distributed or median with interquartile ranges (IQR) if the data were not normally distributed. Categorical variables were presented as frequency/counts and percentage. The Charlson comorbidity index (CCI) was calculated using the method described by Deyo et al. [10] We presented the trends in bariatric procedures on a quarterly basis from 2000 to 2020, describing the procedure types by both percentages of all procedure types and also as volume of procedure performed.

We used inverse probability of treatment weights (IPT) to control for confounding. We estimated cumulative incidence of complications of SG, RYGB and BPD/DS at 30-days, 6-months, and 1-year postoperatively using weighted Kaplan–Meier analyses. We estimated the weights based on the inverse of predicted probability (propensity scores) of receiving SG vs. RYGB or BPD/DS vs. RYGB using two separate logistic regression models. These propensity score models included patient age, sex, all comorbidities in CCI (excluding human immunodeficiency virus), location of the procedure (inpatient or outpatient), and the year of the index procedure. The IPT weights were stabilized using the marginal probability of receiving SG vs. RYGB or BPD/DS vs. RYGB. We calculated the adjusted Hazard ratio (aHR) comparing SG vs. RYGB and BPD/DS vs. RYGB on the 1-year risk of complications from 2010 to 2020 with weighted Kaplan–Meier analyses and 95% confidence intervals (CIs) using robust-variance estimators. Similarly, we calculated weighted cumulative incidence of procedure-specific revisions between SG and RYGB at 6-months and 1-year using weighted survival models and calculated aHR, 95% CIs (using robust-variance estimators). All analyses were performed using SAS 9.4 (SAS Inc., Cary, NC).

## Results

We identified 343,727 unique individuals who underwent one or more bariatric procedures between 2000 and 2020 and met our inclusion criteria. The baseline characteristics were stratified by procedure type and described in Table 1. The mean age was 43.5 ± 10.6 years old, with the cohort consisting of 78.9% female patients and 21.1% male patients. Laparoscopic RYGB and BPD/DS patients had a higher prevalence of diabetes mellitus at baseline when compared to SG (37.1% and 37.5% vs. 28.6%, respectively). The median length of stay (LOS) was higher in open and laparoscopic RYGB when compared to SG (3 days (IQR: 2–4) and 2 days (1–2) vs. 1 day (1–2)). Table 1 details baseline characteristics of patients by type of index procedure.

**Table 1:** Patient baseline characteristics stratified by procedure type

Procedure Type	Open RYGB	Lap RYGB	SG	GB	VBG	BPD/DS
Total	23,637 (6.76)	114,156 (32.7)	140,209 (40.1)	68,140 (19.5)	1136 (0.33)	2133 (0.61)
Male	4412 (18.7)	24,272 (21.3)	30,211 (21.6)	14,235 (20.89)	205 (18.05)	538 (25.2)
Age (Median, IQR)	45 (36,52)	44 (36,52)	43 (35,51)	43 (35,52)	42 (34,51)	43 (35,51)
CCI (Median, IQR)	0 (0,1)	1 (0,1)	1 (0,1)	0 (0,1)	0 (0,1)	1 (0,2)
Myocardial infarction	176 (0.74)	1111 (0.97)	1265 (0.9)	381 (0.56)	5 (0.44)	20 (0.94)
Congestive heart failure	747 (3.16)	2624 (2.3)	3028 (2.16)	933 (1.37)	31 (2.73)	72 (3.38)
Peripheral vascular disease	153 (0.65)	1641 (1.44)	2611 (1.86)	874 (1.28)	8 (0.7)	26 (1.22)
Cerebrovascular disease	41 (0.17)	512 (0.45)	1256 (0.9)	160 (0.23)	0 (0)	8 (0.38)
Hemiplegia paraplegia	11 (0.05)	83 (0.07)	120 (0.09)	30 (0.04)	0 (0)	3 (0.14)
Dementia	15 (0.06)	35 (0.03)	58 (0.04)	9 (0.01)	0 (0)	0 (0)
COPD	4365 (18.47)	21,360 (18.7)	26,100 (18.6)	9983 (14.65)	166 (14.61)	433 (20.3)
Rheumatoid Arthritis	308 (1.30)	1970 (1.73)	2862 (2.04)	884 (1.3)	18 (1.58)	28 (1.31)
Peptic ulcer disease	52 (0.22)	856 (0.75)	2007 (1.43)	91 (0.13)	9 (0.79)	28 (1.31)
Diabetes mellitus	7244 (30.65)	42,386 (37.1)	40,104 (28.6)	18,671 (27.4)	272 (23.94)	799 (37.5)
Renal disease	238 (1.01)	2204 (1.93)	2826 (2.02)	754 (1.11)	14 (1.23)	50 (2.34)
Liver disease	441 (1.87)	6962 (6.1)	14,526 (10.4)	775 (1.14)	18 (1.58)	312 (14.6)
Cancer	433 (1.83)	2224 (1.95)	3311 (2.36)	1253 (1.84)	27 (2.38)	55 (2.58)
HIV	4 (0.02)	111 (0.1)	294 (0.21)	73 (0.11)	0 (0)	4 (0.19)
Inpatient procedures	20,879 (88.33)	100,670 (88.2)	105,677 (75.4)	12,603 (18.5)	791 (69.63)	1839 (86.2)
LOS (median, IQR)	3 (2,4)	2 (1,2)	1 (1,2)	1 (1,1)	2 (1,3)	2 (1,3)

Numbers reported as n, (%). Interquartile range (IQR), Charlson comorbidity index (CCI), chronic obstructive pulmonary disease (COPD), human immunodeficiency virus (HIV), Length of stay (LOS), Roux-en-y gastric bypass (RYGB), sleeve gastrectomy (SG), adjustable gastric banding (AGB), vertical banded gastroplasty (VBG), biliopancreatic diversion with duodenal switch (BPD/DS)

**Fig. 1:**
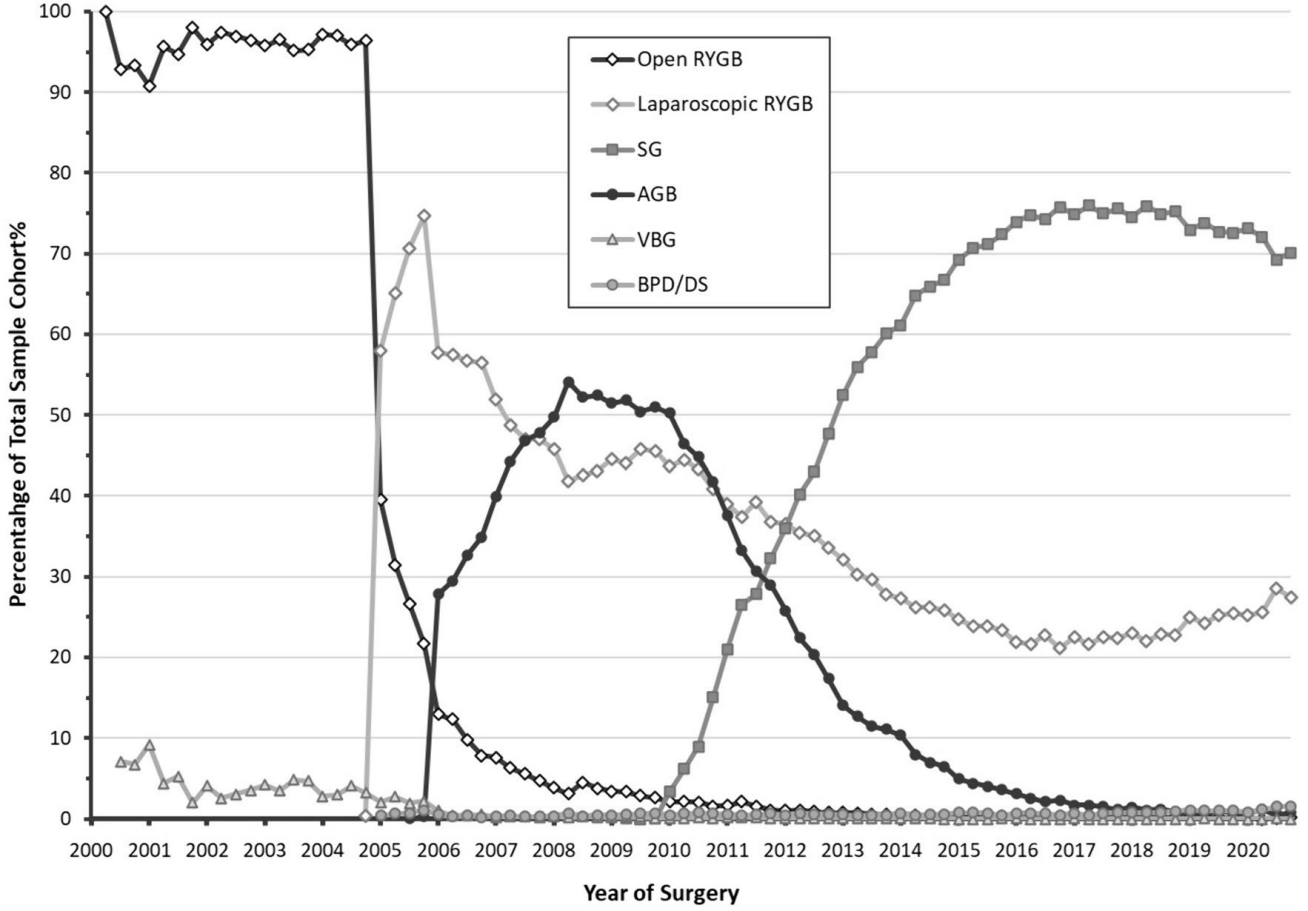
Trend of bariatric surgery 2000–2020, in percentage of total sample cohort. Overall trend of open roux-en-y gastric bypass (RYGB), laparoscopic RYGB, sleeve gastrectomy (SG), laparoscopic adjustable gastric banding (AGD), vertical banded gastroplasty (VBG), and biliopancreatic diversion with duodenal switch (BPD/DS) from 2000 to 2020 in terms of percentage of total sample cohort

**Fig. 2:**
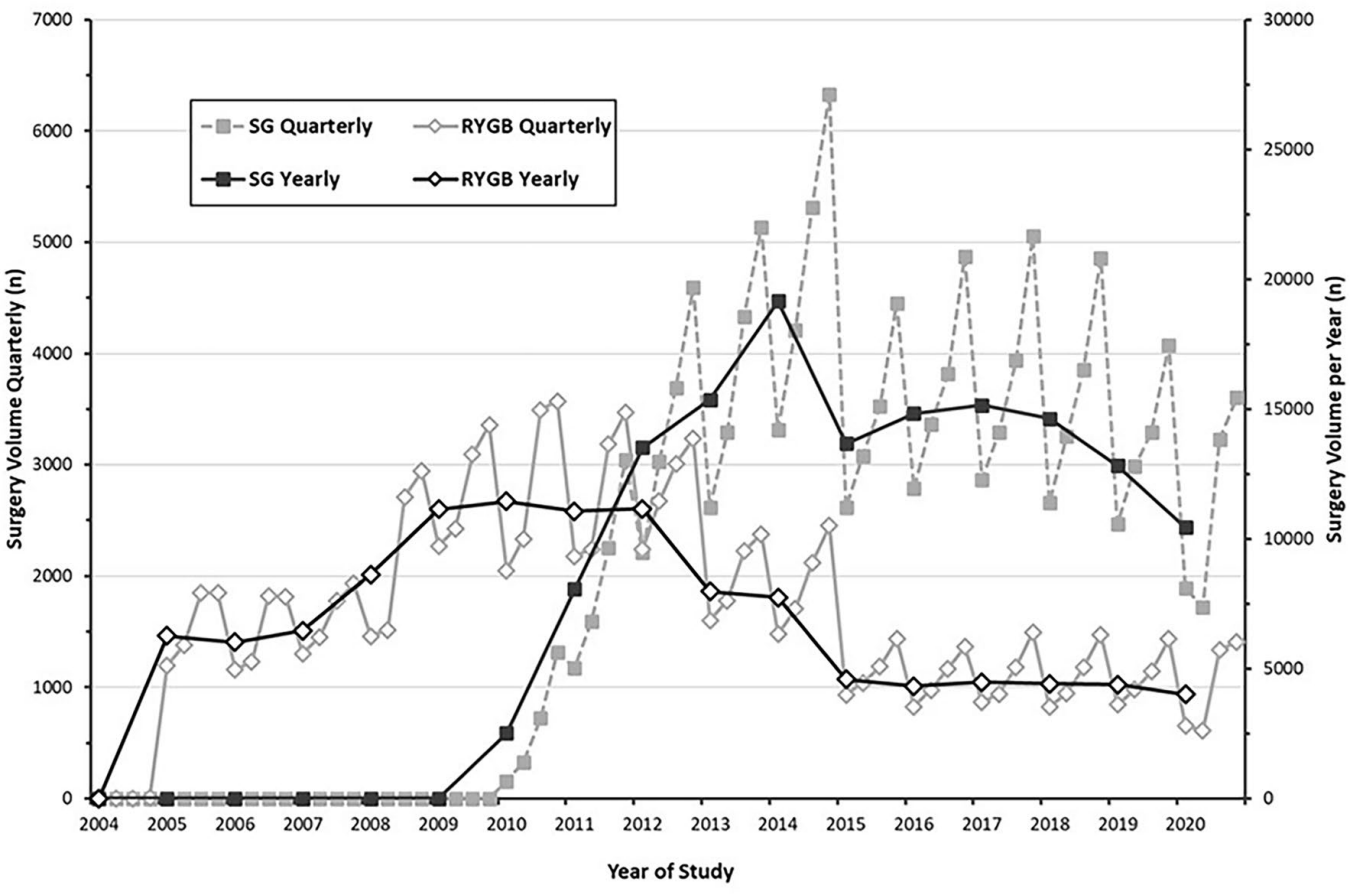
SG and laparoscopic RYGB sample cohort trend 2004–2020. Overall quarterly and yearly trend of laparoscopic roux-en-y gastric bypass (RYGB) and sleeve gastrectomy (SG) in terms of sample volume

The trend of bariatric surgery procedures performed between 2000 and 2020 as a percentage from the total sample cohort is shown in Fig. 1. Open RYGB, AGB, and VBG all dramatically reduced in overall utilization over the past two decades. SG increased in utilization rapidly since 2010 and appeared to have plateaued around 2018 with a small decline thereafter (peak of 75.9% in 2018 to 69.2% in 2020, in percentage of total sample cohort), while laparoscopic RYGB appeared to increase around the same time (22.1% in 2018 to 28.6% in 2020, as a percentage of total sample cohort). Figure 2 demonstrates a sub-analysis of the actual quarterly and annual volume from our cohort for SG and Laparoscopic RYGB from 2004 to 2020. When examining the trend of bariatric surgery in terms of actual volume from the total sample cohort, however, the laparoscopic RYGB volume appeared to hold steady after 2015 while SG, again, appeared to decline beginning in 2018.

The risks of complications between SG and laparoscopic RYGB, and between laparoscopic RYGB and BPD/DS, at 1-year postoperatively are reported in Tables 2 and 3, respectively. Compared to laparoscopic RYGB, SG was associated with lower 1-year incidence for readmission, emergency room visit, wound dehiscence, dehydration, weight/feeding disorder, malabsorption, anemia, vitamin deficiency, nausea or vomiting, dysphagia, other digestive symptoms, pneumonia, sepsis, urinary complications, gastrointestinal ulcers, obstruction, hemorrhage/hematoma, gallbladder disorders, and acute renal failure. Conversely, compared to laparoscopic RYGB, SG was associated with higher 1-year incidence of esophagogastroduodenoscopy (EGD), heartburn, gastritis, portal vein thrombosis, and hernias of all types. Compared to laparoscopic RYGB, BPD/DS was associated with higher incidence of readmission, heartburn, and gastritis. Conversely, BPD/DS was associated with a lower incidence of EGD and gastrointestinal ulcer when compared to laparoscopic RYGB.

**Table 2:** SG vs. laparoscopic RYGB

	SG			RYGB			SG VS. RYGB	
	1-mo	6-mo	12-mo	1-mo	6-mo	12-mo	aHR, 95% CI	p-values
Healthcare utilization								
Readmission (all cause)	3.44	6.68	10.20	5.18	10.45	14.98	**0.65 (0.64, 0.68)**	** < 0.0001**
30-day readmission*	3.44			5.18			**0.65 (0.62, 0.69)**	** < 0.0001**
Emergency room visit	8.98	19.84	29.15	12.71	26.99	37.85	**0.72 (0.70, 0.73)**	** < 0.0001**
EGD	27.87	29.81	31.20	22.17	29.18	32.17	**1.13 (1.11, 1.15)**	** < 0.0001**
Bariatric surgery								
Leak	0.01	0.01	0.01	0.01	0.01	0.01	1.26 (0.51, 3.12)	0.610
Wound dehiscence	0.73	1.05	1.33	1.41	1.86	2.22	**0.58 (0.54, 0.63)**	** < 0.0001**
Digestive complications								
Dehydration	5.81	8.95	10.36	6.84	11.80	13.66	**0.75 (0.73, 0.78)**	** < 0.0001**
Weight/feeding disorder	1.94	7.51	11.10	1.68	8.54	13.44	**0.83 (0.81, 0.86)**	** < 0.0001**
Malabsorption	3.51	18.94	25.19	7.66	32.89	43.25	**0.51 (0.50, 0.52)**	** < 0.0001**
Anemia	9.19	19.51	26.29	11.29	23.13	31.06	**0.82 (0.80, 0.83)**	** < 0.0001**
Vitamin deficiency	9.68	31.68	42.58	11.37	36.07	48.78	**0.84 (0.83, 0.85)**	** < 0.0001**
Nausea/Vomiting	7.95	13.11	16.40	8.99	18.56	23.06	**0.70 (0.69, 0.72)**	** < 0.0001**
Heartburn	1.10	1.99	2.69	0.93	1.46	1.91	**1.38 (1.28, 1.49)**	** < 0.0001**
Dysphagia	2.49	4.45	5.27	2.97	8.16	9.48	**0.55 (0.53, 0.57)**	** < 0.0001**
Gastritis	19.64	21.16	22.37	2.65	5.17	7.18	**4.28 (4.14, 4.44)**	** < 0.0001**
Other digestive symptoms	2.95	5.16	7.26	4.53	9.42	12.44	**0.56 (0.55, 0.58)**	** < 0.0001**
Cardiovascular complications								
Acute MI/Angina*	0.33			0.39			**0.84 (0.72, 1.00)**	**0.039**
Stroke/TIA*	0.20			0.18			1.06 (0.85, 1.34)	0.595
Pulmonary embolism	0.49	0.77	0.91	0.60	0.86	1.03	**0.88 (0.79, 0.97)**	**0.014**
Deep venous thrombosis	1.10	1.79	2.20	1.14	1.89	2.32	0.95 (0.88, 1.01)	0.119
Portal vein thrombosis	0.19	0.22	0.24	0.03	0.05	0.07	**3.93 (2.82, 5.48)**	** < 0.0001**
Infections								
Pneumonia*	0.71			1.20			**0.58 (0.52, 0.65)**	** < 0.0001**
Sepsis*	0.40			0.66			**0.60 (0.52, 0.69)**	** < 0.0001**
Urinary complications	2.82	7.04	11.11	3.62	9.14	13.99	**0.78 (0.75, 0.80)**	** < 0.0001**
Gastrointestinal complications								
Gastrointestinal ulcer	0.43	0.78	0.99	0.90	3.77	5.34	**0.19 (0.18, 0.21)**	** < 0.0001**
Intestinal obstruction	0.95	1.21	1.43	2.04	2.96	3.64	**0.40 (0.37, 0.43)**	** < 0.0001**
Gastrointestinal hemorrhage	0.77	1.29	1.79	1.92	3.12	4.06	**0.43 (0.40, 0.46)**	** < 0.0001**
Other complications								
Liver necrosis	0.05	0.11	0.14	0.06	0.10	0.16	0.93 (0.71, 1.20)	0.566
Gall bladder disorders	3.17	5.37	7.87	4.46	7.34	10.50	**0.73 (0.71, 0.76)**	** < 0.0001**
Pancreatic disorders	0.41	0.68	1.00	0.38	0.74	1.10	0.92 (0.82, 1.02)	0.102
Acute renal failure*	0.86			1.08			**0.79 (0.71, 0.87)**	** < 0.0001**
Neuromuscular complications	0.40	1.69	3.15	0.45	1.98	3.79	**0.83 (0.78, 0.88)**	** < 0.0001**
Skin Symptoms complications	3.74	9.00	14.33	4.24	10.36	16.38	**0.87 (0.84, 0.89)**	** < 0.0001**
Hemorrhage/hematoma	0.82	0.94	1.08	1.42	1.59	1.80	**0.59 (0.54, 0.64)**	** < 0.0001**
Hernias of all types	25.45	26.74	28.16	19.13	21.03	23.22	**1.36 (1.34, 1.39)**	** < 0.0001**

Bold values indicate that statistically significant P-values < 0.05

2010–2020 Standardized postoperative complication weighted risks at 1, 6, and 12 months

Numbers reported as weighted risks (%) for 1-month, 6-months, and 12-months postoperative complications from 2010 to 2020. Confidence intervals (CI), adjusted Hazard ratio (aHR), Sleeve gastrectomy (SG), Roux-en-y gastric bypass (RYGB), Month (mo), Esophagogastroduodenoscopy (EGD), Myocardial infarction (MI), Transient ischemic attack (TIA)

^*^Only 1-month outcome reported postoperatively

**Table 3:** BPD/DS vs. laparoscopic RYGB

	BPD/DS			RYGB			BPD/DS Vs. RYGB	
	1-mo	6-mo	12-mo	1-mo	6-mo	12-mo	aHR, 95% CI	p-values
Healthcare utilization								
Readmission (all cause)	8.32	15.24	20.17	5.05	10.68	15.51	**1.38 (1.21, 1.57)**	** < .0001**
30-day readmission*	8.32			5.05			**1.67 (1.38, 2.02)**	** < .0001**
Emergency room visit	17.15	31.57	43.07	12.38	26.52	37.35	**1.24 (1.13, 1.35)**	** < .0001**
EGD	21.44	26.25	28.06	22.48	29.61	32.59	**0.86 (0.78, 0.95)**	**0.003**
Digestive complications								
Dehydration	12.09	19.27	23.08	7.15	12.38	14.41	**1.67 (1.48, 1.87)**	** < .0001**
Weight/feeding disorder	6.37	13.68	19.14	1.77	8.72	13.69	**1.55 (1.35, 1.77)**	** < .0001**
Malabsorption	4.04	31.14	43.52	7.62	32.81	43.18	0.95 (0.87, 1.03)	0.227
Anemia	12.79	27.75	39.97	11.25	23.22	31.18	**1.30 (1.20, 1.42)**	** < .0001**
Vitamin deficiency	12.09	40.89	56.00	10.79	35.04	47.46	**1.23 (1.14, 1.32)**	** < .0001**
Nausea/Vomiting	13.11	21.79	25.97	8.58	17.96	22.31	**1.23 (1.10, 1.37)**	** < .0001**
Heartburn	1.91	3.05	4.43	0.75	1.21	1.61	**2.72 (2.13, 3.49)**	** < .0001**
Dysphagia	4.29	7.93	9.86	2.79	7.92	9.21	1.08 (0.91, 1.28)	0.408
Gastritis	16.09	17.99	19.77	2.48	4.94	6.86	**3.80 (3.36, 4.30)**	** < .0001**
Other digestive symptoms	1.45	2.06	2.28	0.69	1.57	1.78	1.30 (0.94, 1.79)	0.109
Cardiovascular complications								
Acute MI/Angina*	0.71			0.44			1.63 (0.88, 3.01)	0.123
Stroke/TIA*	0.19			0.19			1.00 (0.29, 3.41)	1.000
Pulmonary embolism	0.73	1.57	1.57	0.62	0.91	1.08	1.49 (0.98, 2.27)	0.064
Deep venous thrombosis	1.99	3.59	4.30	1.23	2.05	2.49	**1.74 (1.32, 2.29)**	** < .0001**
Portal vein thrombosis	0.12	0.12	0.15	0.03	0.05	0.07	2.30 (0.81, 6.53)	0.119
Infections								
Pneumonia*	2.76			1.20			**2.34 (1.67, 3.29)**	** < .0001**
Sepsis*	2.04			0.66			**3.36 (2.33, 4.84)**	** < .0001**
Gastrointestinal complications								
Gastrointestinal ulcer	1.52	2.36	3.06	1.01	4.16	5.82	**0.55 (0.40, 0.75)**	** < .0001**
Intestinal obstruction	3.17	4.08	5.41	2.50	3.66	4.51	1.19 (0.92, 1.53)	0.19
Gastrointestinal hemorrhage	2.57	2.57	4.01	3.17	3.17	4.18	0.94 (0.71, 1.24)	0.647
Other complications								
Liver necrosis	0.32	0.40	0.48	0.07	0.12	0.17	**3.08 (1.33, 7.14)**	**0.009**
Gall bladder disorders	24.41	26.52	28.53	4.56	7.39	10.52	**4.05 (3.65, 4.49)**	** < .0001**
Pancreatic disorders	1.58	2.17	2.54	0.39	0.76	1.14	**2.41 (1.69, 3.44)**	** < .0001**
Acute renal failure*	3.05			1.15			**2.68 (1.93, 3.72)**	** < .0001**
Neuromuscular complications	0.84	2.62	4.51	0.42	1.85	3.45	**1.35 (1.03, 1.77)**	**0.031**
Skin Symptoms complications	5.25	13.85	21.11	4.31	10.23	15.90	**1.37 (1.21, 1.55)**	** < .0001**
Hemorrhage/hematoma	2.30	2.36	2.58	1.55	1.73	1.96	1.35 (0.97, 1.89)	0.074
Hernias of all types	17.95	20.94	26.55	18.69	20.54	22.75	1.10 (0.99, 1.22)	0.064

Bold values indicate that statistically significant P-values < 0.05

2010–2020 Standardized postoperative complication weighted risks at 1, 6, and 12 months

Numbers reported as weighted risks (%) for 1-month, 6-months, and 12-months postoperative complications from 2010 to 2020. Confidence intervals (CI), adjusted Hazard ratio (aHR), Biliopancreatic diversion with duodenal switch (BPD/DS), Roux-en-y gastric bypass (RYGB), Month (mo), Esophagogastroduodenoscopy(EGD), Myocardial infarction (MI), Transient ischemic attack (TIA)

^*^Only 1-month outcome reported postoperatively

Table 4 shows the comparison of revision volumes for the sample cohort over 2000–2020. There were a total of 5370 patients with revisions or conversions captured within our cohort with 151 patients undergoing a second revision or conversion. Overall, the most common revisions were ABG to SG (2220), ABG to RYGB (1092), SG to RYGB (819), and AGB revisions (399). SG was compared with laparoscopic RYGB in Table 5 in terms of incidence of general surgical interventions at 6-months and 1-year. SG had a significantly lower 1-year incidence of need for surgical intervention when compared to RYGB. The 1-year incidence of leak, perforation, or ulcer-related complication was not significantly different between SG and RYGB.

**Table 4:** Revision types and volume comparison for SG, RYGB, and AGB

Revision type		Number of revisions (% of total volume)
Primary SG		136,483 (100)
SG Revisions	SG Revisions	200 (0.15)
	SG to RYGB	819 (0.60)
	SG to BPD/DS	74 (0.05)
Primary RYGB		111,595 (100)
RYGB Revisions	RYGB Revisions	193 (0.17)
	RYGB to SG	39 (0.03)
	RYGB to BPD/DS	6 (0.01)
Primary AGB		63,646 (100)
AGB Revisions	AGB Revisions	399 (0.63)
	AGB to SG	2220 (3.49)
	AGB to RYGB	1092 (1.72)
	AGB to BPD/DS	19 (0.03)

Sleeve gastrectomy (SG), Roux-en-y gastric bypass (RYGB), Biliopancreatic diversion with duodenal switch (BPD/DS), Adjustable gastric banding (AGB)

**Table 5:** Comparison of cumulative incidences of general surgical interventions for SG versus RYGB at 6-months and 1-year postoperatively

Other Non-Revisional Surgical Interventions	6-months		1-year		1-year aHR, 95% CI	
	RYGB	SG	RYGB	SG	aHR	p-value
General unlisted laparoscopic procedures on the intestine or stomach (CPTs 44,238, 43,999, 43,659)	1.96	1.74	2.44	1.81	**0.79 (0.74, 0.85)**	** < .0001**
Postoperative perforation, leak, and ulcer related (CPTs 43,610, 43,840, 49,905, 49,329)	2.68	2.70	2.90	2.78	0.98 (0.92, 1.04)	0.5228
Internal hernia or bowel obstruction (CPTs 44,050, 44,180, 44,850)	2.97	1.55	3.64	1.65	**0.47 (0.44, 0.51)**	** < .0001**

Bold values indicate that statistically significant P-values < 0.05

Numbers reported as weighted risks (%) for 6-months, and 1-year postoperative complications. Adjusted hazard ratio (aHR) Confidence intervals (CI), Roux-en-y gastric bypass (RYGB), Sleeve gastrectomy (SG)

## Discussion

This study evaluated the trends of bariatric surgery utilization, postoperative complications, conversions and revisions in the last 20 years using a national commercial claims database. We compared the incidence of postoperative complications between SG and laparoscopic RYGB as well as between laparoscopic RYGB and BPD/DS. Compared to laparoscopic RYGB, SG was associated with lower incidences of most postoperative complications in general, but higher incidences of heartburn, gastritis, portal vein thrombosis, hernias of all types, and need for EGD. These findings are consistent with the most recent studies where SG is often found to have a comparable or lower incidence of postoperative complication than RYGB but is limited by an increased incidence of GERD, worsening of GERD symptoms, or esophagitis [11–13]. One of the hypotheses for GERD after SG is an increase in intragastric pressure as well as gastroesophageal pressure gradient, resulting in increased acid exposure and number of reflux episodes [14]. The higher incidences of EGD in SG patients 1-year postoperatively could potentially be secondary to the need for evaluation of gastrointestinal symptoms from unrecognized esophageal dysmotility, GERD, postoperative nausea and vomiting (PONV), or stenosis of the sleeve. A recent study by Zhu et al. showed that, compared to RYGB, SG is associated with a higher incidence of PONV (77.4% versus 21.5%), especially in patients with preoperative reflux symptoms [15]. These symptoms after SG can often be signs of developing or worsening GERD and should be treated and monitored accordingly. Our study showed a higher 1-year incidence of hernias of all types in SG when compared to laparoscopic RYGB. This finding could potentially be due to increased attention to the hiatus during SG leading to increased diagnosis and aggressive treatment of hiatal hernias. The need for gastric specimen extraction in SG also could lead to potential incisional hernias depending on extraction site and port closure methods. The risk of incisional hernia after sleeve gastrectomy was found by Ahlqvist et al. to be as high as 21.5% when using a Hasson technique and continuous closure of the port site [16]. Unfortunately, we did not differentiate between various hernia types in our study. Further study and sub-analysis are planned to investigate the incidence of hiatal hernias and incisional hernias in the SG and RYGB populations.

When compared to laparoscopic RYGB, we found BPD/DS was associated with higher 1-year incidences of postoperative complications in general but lower incidences of EGD and gastrointestinal ulcer. This finding is consistent with other studies comparing RYGB to BPD/DS, where BPD/DS is associated with a higher incidence of early 30-days complications (15.3% vs. 8.1%) and long-term adverse events (2.7 vs. 0.9 events per patient) up to 15 years post-operatively [17, 18]. Our reported BPD/DS results based on the 43,845 CPT code are limited as it is likely a combination of laparoscopic and open approaches. Open BPD/DS, though rarely performed, is often associated with higher incidence of postoperative adverse events and longer LOS. Yet, studies that isolated and compared the laparoscopic approaches of RYGB and BPD/DS also showed higher incidence of adverse events for BPD/DS [19, 20]. Although not specifically identified in our current study due to a lack of a discrete CPT code, single anastomosis duodeno-ileal bypass with sleeve gastrectomy (SADI-S) is a newer procedure aimed at reducing the operative and perioperative complications of BPD/DS while preserving its remarkable weight loss capabilities and effect on comorbidities. Current studies are mixed regarding if SADI-S is comparable in terms of perioperative complications to RYGB and more long-term studies are required to determine its role among the current bariatric armamentarium [21–23].

Among the revisions and conversions, the highest volumes of conversions were from AGB to SG and AGB to RYGB. The third most common conversion within our cohort was SG to RYGB. This volume reflected the conversions over the span of 2000–2020, which had a higher number of AGB conversions when compared to a more recent Metabolic and Bariatric Surgery Accreditation and Quality Improvement Program (MBSAQIP) retrospective analysis of conversions performed in 2020 [24].This MBSAQIP study found that the most converted index operations were SG (49.3%) and AGB (45.9%), with the most frequent conversion being SG to RYGB (40.3%) and AGB to SG (27.3%) [24]. This change in proportions of conversions when compared to our study is likely due to the tremendous decline in AGB, which now comprises less than 1% of bariatric procedures. With SG being the most common bariatric procedure in more recent years, it is no surprise that SG to RYGB has become the most common conversion. While our study could not specifically examine the indications for revisions or conversions, most studies identify GERD as the main reason for SG conversions and report a 2–6% rate of SG to RYGB conversion due to severe reflux alone [24, 25]. Inadequate weight loss or weight regain are also indications for SG to RYGB conversions but may potentially be underrepresented given the frequent lack of insurance coverage for these indications. In our study, the proportion of SG to RYGB conversion was only 0.6% out of the total number of SG captured and the overall volume of conversions and revisions were lower than most database studies [24, 26]. We suspect this was due to the limitation of using CPT codes and a private claims database, where change in employer and insurance will lead to a loss of follow up and inability to capture subsequent revisions and conversions due to lack of continuous enrollment.

When comparing 6-months and 1-year surgical interventions (including unlisted procedures, omental patch, internal hernia, and bowel obstructions), SG had a significantly lower incidence of need for surgical intervention by 1-year when compared to RYGB, consistent with prior literature [27]. However, this increased risk for RYGB was not seen when comparing the rates of intervention for leak, perforation, or ulcer. This is contrary to most studies but may be due to only having up to 1 year of follow up [28]. Another potential reason may be that the study period covers the popularization of SG and there may be higher leak rates during the learning curve that offset the leak rates in more recent times [29]. Our results were also limited due to the use of CPT codes as there may be other procedure codes used for leaks that were not reported or captured accurately. In our study, RYGB was associated with a higher 1-year incidence of lysis of adhesions, reduction of volvulus, and closure of internal hernia or mesenteric defect. This was consistent with other reports and is an inherent risk of intestinal reconstruction [30]. Overall, the postoperative complications from this MarketScan database study appear consistent with current literature but were limited by intrinsic limitations of utilizing administrative claims databases.

Laparoscopic SG continued to be the most commonly performed bariatric procedure, but the observed volume in our cohort appeared to decline in 2018. This decline in volume, also seen in the rest of the cohorts for other commonly performed bariatric procedures, was in contrast to data from other national samples such as the MBSAQIP and Medicare and Medicaid databases, where an increase in overall volume was demonstrated until the reduction in 2020 secondary to the COVID-19 pandemic [6, 31]. The decline in volume within the MarketScan database may potentially be due to the limitation that the sampled volume is based on private employer insurance, where changes in bariatric surgery coverage, preoperative requirements, and employment can affect access to care [32, 33]. Interestingly, the decline in RYGB volume appeared less precipitous than the decline in SG. This resulted in an overall increase in proportion of RYGB performed and a decrease in proportion of SG performed since around 2018, although both volumes were decreasing. This increase in proportion of RYGB in recent years was also seen in the most recent MBSAQIP analysis, where RYGB increased to 22.2% from 17% and SG decreased to 57.4% from 61.4% from 2018 to 2022 [34]. This rebound of RYGB utilization in recent years may signify the growing recognition of the limitations of SG. Currently, there is no established consensus on the choice of SG vs. RYGB other than in patients with severe GERD and patients with type-II diabetes mellitus despite numerous randomized trials comparing the two and a recent expert modified Delphi consensus [35]. Although both procedures produce excellent weight loss and comorbidity reduction results, the choice of the procedure ultimately is dependent on patient characteristics, patient preference, and surgeon experience as the two procedures have differing profiles of benefits and risks associated with them.

Our study was limited due to its retrospective nature and its restriction to private, employer-sponsored insurance claims. First, the procedures and complications were abstracted using CPT and ICD codes (ICD-9 was used until 2015, then followed by ICD-10), which rely on healthcare providers’ and coders’ accurate input and may have led to misclassification of some of the complications. Second, patients who switch insurance during the study period are lost to follow up within the database, likely contributing to the low incidence of revisions captured in our study. Third, the database does not include information on death, so we could not account for death as a competing risk in our analysis. Future studies using the MBSAQIP, the Michigan Bariatric Surgery Collaborative, or other statewide databases may be helpful in evaluating whether payor types affect the rate of revisions. Despite these limitations, this study is one of the largest longitudinal studies on the trends of bariatric surgery based on private claims data. The MarketScan claims database was chosen specifically for its ability to track individuals longitudinally regardless of which healthcare system to which they present with complications or revisions. This capability provides a unique perspective over traditional databases where complications or revisions can be missed if patients present to a different healthcare system. This study also demonstrated validity of the private claims database with results consistent with other published retrospective and prospective studies. We plan to utilize the strengths of the private claims database to investigate the prescription patterns and trends of Glucagon-like peptide-1 agonist medications as well as use of proton pump inhibitors in the preoperative and postoperative settings in the cohort identified in this study.

In conclusion, this IBM MarketScan Commercial database study from 2000 to 2020 demonstrated a reduction in SG utilization starting from 2018 with a corresponding increase in proportion of RYGB performed, which matches well with recent MBSAQIP data. Although SG remains the most frequently utilized bariatric procedure given its low risks for complications, the incidence of GERD, weight regain, and potential need for conversion may limit its use in certain populations. RYGB continues to be one of the gold standard bariatric procedures and should remain part of the modern bariatric surgeon’s armamentarium.

### Supplementary Information

Below is the link to the electronic supplementary material.

Supplementary file1 (DOCX 24 KB)

## Data Availability

Source data: Copyright © 2021 IBM Watson Health. All Rights Reserved. IBM Watson Health and MarketScan are trademarks of IBM Corporation in the United States, other countries or both.
