# Supplementary material for: Bariatric surgery: trends in utilization, complications, conversions and revisions
Source: Surg Endosc. 2024 Jun 20;38(8):4613–23. doi: 10.1007/s00464-024-10985-7 (PMC11289040; doi:10.1007/s00464-024-10985-7)
Supplement: Supplementary file 1 — Supplementary file1 (DOCX 24 KB) [file 464_2024_10985_MOESM1_ESM.docx]

## **Appendix 1**

**ICD9 and ICD 10 codes**

| **Diagnosis** | **ICD-9** | **ICD-10** |
| --- | --- | --- |
| Dehydration | 276 | E860 |
| Pain | 338,7890,7896 | G891, G8918, G8928, G8929, G894, R100, R1010, R1011, R1012, R1013, R1030, R1031, R1032, R1033, R10811, R10812, R10813, R10814, R10815, R10816, R10817, R10819, R10821, R10822, R10823, R10824, R10825, R10826, R10827, R10829, R1084, R109, R52 |
| MI complications | 410,411,413 | I200, I201, I208, I209, I21, I2101, I2102, I2109, I2111, I2119, I2121, I2129, I213, I214, I219, I21A1, I21A9, I220, I221, I222, I228, I229, I240, I241, I248, I249 |
| Pulmonary embolism | 4151 | I26, I260, I2601, I2602, I2609, I269, I2690, I2692, I2693, I2694, I2699 |
| Stroke | 434,435,436 | G450 , G451 , G452 , G458 , G459 , G460 , G461 , G462 , I6330 , I63311 , I63312 , I63313 , I63319 , I63321 , I63322 , I63323 , I63329 , I63331 , I63332 , I63333 , I63339 , I63341 , I63342 , I63343 , I63349 , I6339 , I6340 , I63411 , I63412 , I63413 , I63419 , I63421 , I63422 , I63423 , I63429 , I63431 , I63432 , I63433 , I63439 , I63441 , I63442 , I63443 , I63449 , I6349 , I6350 , I63511 , I63512 , I63513 , I63519 , I63521 , I63522 , I63523 , I63529 , I63531 , I63532 , I63533 , I63539 , I63541 , I63542 , I63543 , I63549 , I6359 , I636 , I638 , I639 , I6601 , I6602 , I6603 , I6609 , I6611 , I6612 , I6613 , I6619 , I6621 , I6622 , I6623 , I6629 , I663 , I668 , I669 , I67841 , I67848 , I6789 |
| DVT | 451,453 | I82210 , I82211 , I82220 , I82221 , I82290 , I82291 , I823 , I82401 , I82402 , I82403 , I82409 , I82411 , I82412 , I82413 , I82419 , I82421 , I82422 , I82423 , I82429 , I82431 , I82432 , I82433 , I82439 , I82441 , I82442 , I82443 , I82449 , I82491 , I82492 , I82493 , I82499 , I824Y1 , I824Y2 , I824Y3 , I824Y9 , I824Z1 , I824Z2 , I824Z3 , I824Z9 , I82501 , I82502 , I82503 , I82509 , I82511 , I82512 , I82513 , I82519 , I82521 , I82522 , I82523 , I82529 , I82531 , I82532 , I82533 , I82539 , I82541 , I82542 , I82543 , I82549 , I82591 , I82592 , I82593 , I82599 , I825Y1 , I825Y2 , I825Y3 , I825Y9 , I825Z1 , I825Z2 , I825Z3 , I825Z9 , I82601 , I82602 , I82603 , I82609 , I82611 , I82612 , I82613 , I82619 , I82621 , I82622 , I82623 , I82629 , I82701 , I82702 , I82703 , I82709 , I82711 , I82712 , I82713 , I82719 , I82721 , I82722 , I82723 , I82729 , I82811 , I82812 , I82813 , I82819 , I82890 , I82891 , I8290 , I8291 , I82A11 , I82A12 , I82A13 , I82A19 , I82A21 , I82A22 , I82A23 , I82A29 , I82B11 , I82B12 , I82B13 , I82B19 , I82B21 , I82B22 , I82B23 , I82B29 , I82C11 , I82C12 , I82C13 , I82C19 , I82C21 , I82C22 , I82C23 , I82C29 |
| Portal vein thrombosis | 452 | I81 |
| Hypotension | 458 | I81, 458, I951, I952, I953, I9581, I9589, I959 |
| Pneumonia | 480,481,482, 483,484,485,486,99731,99732 | A221 , A3701 , A3711 , A3781 , A3791 , A481 , B250 , B440 , B7781 , J120 , J121 , J122 , J123 , J1281 , J1289 , J129 , J13 , J14 , J150 , J151 , J1520 , J15211 , J15212 , J1529 , J153 , J154 , J155 , J156 , J157 , J158 , J159 , J160 , J168 , J17 , J180 , J181 , J188 , J189 , J95851 , J9589 |
| GI ulcer | 531,532,533,534 | K250 , K251 , K252 , K253 , K254 , K255 , K256 , K257 , K259 , K260 , K261 , K262 , K263 , K264 , K265 , K266 , K267 , K269 , K270 , K271 , K272 , K273 , K274 , K275 , K276 , K277 , K279 , K280 , K281 , K282 , K283 , K284 , K285 , K286 , K287 , K289 |
| Gastritis | 535 | K2900 , K2901 , K2920 , K2921 , K2930 , K2931 , K2940 , K2941 , K2950 , K2951 , K2960 , K2961 , K2970 , K2971 , K2980 , K2981 , K2990 , K2991 , K5281 |
| Stomach complications | 536,537 | K30 , K310 , K311 , K312 , K313 , K314 , K315 , K316 , K31811 , K31819 , K3182 , K3183 , K3184 , K3189 , K319 , K9420 , K9421 , K9422 , K9423 , K9429 |
| Bariatric complications | 539 | K9501 , K9509 , K9581 , K9589 |
| Hernia | 550,551,552,553 | K4000 , K4001 , K4010 , K4011 , K4020 , K4021 , K4030 , K4031 , K4040 , K4041 , K4090 , K4091 , K4100 , K4101 , K4110 , K4111 , K4120 , K4121 , K4130 , K4131 , K4140 , K4141 , K4190 , K4191 , K420 , K421 , K429 , K430 , K431 , K432 , K433 , K434 , K435 , K436 , K437 , K439 , K440 , K441 , K449 , K450 , K451 , K458 , K460 , K461 , K469 |
| Vascular insufficiency | 557 | K55011 , K55012 , K55019 , K55021 , K55022 , K55029 , K55031 , K55032 , K55039 , K55041 , K55042 , K55049 , K55051 , K55052 , K55059 , K55061 , K55062 , K55069 , K551 , K5530 , K5531 , K5532 , K5533 , K558 , K559 |
| Adhesions | 560 | K5650, K5651, K5652 |
| Nausea and vomiting | 5643, 7870 | K910 , R110 , R1110 , R1111 , R1112 , R1114 , R112 |
| Other digestive | 5644 | K9189 |
| Peritonitis | 567,568,569 | K5700 , K5701 , K5720 , K5721 , K5740 , K5741 , K5780 , K5781 , K630 , K631 , K632 , K633 , K634 , K6381 , K6389 , K639 , K650 , K651 , K652 , K653 , K654 , K658 , K659 , K660 , K661 , K668 , K669 , K67 , K6812 , K6819 , K689 , K91850 , K91858 , K9289 , K929 |
| Liver necrosis | 570 | K7200, K7201, K762 |
| Gall stones | 574,575 | K8000 , K8001 , K8010 , K8011 , K8012 , K8013 , K8018 , K8019 , K8020 , K8021 , K8030 , K8031 , K8032 , K8033 , K8034 , K8035 , K8036 , K8037 , K8040 , K8041 , K8042 , K8043 , K8044 , K8045 , K8046 , K8047 , K8050 , K8051 , K8060 , K8061 , K8062 , K8063 , K8064 , K8065 , K8066 , K8067 , K8070 , K8071 , K8080 , K8081 , K810 , K811 , K812 , K819 , K820 , K821 , K822 , K823 , K824 , K828 , K829 |
| Pancreatic complications | 577 | B252 , K8500 , K8501 , K8502 , K8510 , K8511 , K8512 , K8520 , K8521 , K8522 , K8530 , K8531 , K8532 , K8580 , K8581 , K8582 , K8590 , K8591 , K8592 , K860 , K861 , K862 , K863 , K8681 , K8689 , K869 , K87 |
| GI bleeding | 578 | K920, K921, K922 |
| Malabsorption | 579 | K902 , K903 , K9041 , K9049 , K9089 , K909 , K912 |
| Nutrition deficiency | 260,261,262,263, 264,265,266,267, 268,269 | D81818 , D81819 , E40 , E41 , E42 , E43 , E440 , E441 , E45 , E46 , E500 , E501 , E502 , E503 , E504 , E505 , E506 , E507 , E508 , E509 , E5111 , E5112 , E512 , E518 , E519 , E52 , E530 , E531 , E538 , E539 , E54 , E550 , E559 , E560 , E561 , E568 , E569 , E58 , E59 , E60 , E610 , E611 , E612 , E613 , E614 , E615 , E616 , E617 , E618 , E619 , E630 , E631 , E638 , E639 , E640 , E641 , E642 , E643 , E648 , E649 , M830 , M831 , M832 , M833 , M834 , M835 , M838 , M839 |
| Anemia | 280,281,282,283, 284,285,286,287, 288,289 | D474 , D500 , D501 , D508 , D509 , D510 , D511 , D512 , D513 , D518 , D519 , D520 , D521 , D528 , D529 , D530 , D531 , D532 , D538 , D539 , D550 , D551 , D552 , D553 , D558 , D559 , D560 , D561 , D562 , D563 , D564 , D565 , D568 , D569 , D5700 , D5701 , D5702 , D571 , D5720 , D57211 , D57212 , D57219 , D573 , D5740 , D57411 , D57412 , D57419 , D5780 , D57811 , D57812 , D57819 , D580 , D581 , D582 , D588 , D589 , D590 , D591 , D592 , D593 , D594 , D595 , D596 , D598 , D599 , D600 , D601 , D608 , D609 , D6101 , D6109 , D611 , D612 , D613 , D61810 , D61811 , D61818 , D6182 , D6189 , D619 , D62 , D630 , D631 , D638 , D640 , D641 , D642 , D643 , D644 , D6481 , D6489 , D649 , D65 , D66 , D67 , D680 , D681 , D682 , D68311 , D68312 , D68318 , D6832 , D684 , D6851 , D6852 , D6859 , D6861 , D6862 , D6869 , D688 , D689 , D690 , D691 , D692 , D693 , D6941 , D6942 , D6949 , D6951 , D6959 , D696 , D698 , D699 , D700 , D701 , D702 , D703 , D704 , D708 , D709 , D71 , D720 , D721 , D72810 , D72818 , D72819 , D72820 , D72821 , D72822 , D72823 , D72824 , D72825 , D72828 , D72829 , D7289 , D729 , D730 , D731 , D732 , D733 , D734 , D735 , D7381 , D7389 , D739 , D740 , D748 , D749 , D750 , D751 , D7581 , D7582 , D7589 , D759 , D761 , D762 , D763 , D77 , D892 , I880 , I881 , I888 , I889 |
| Acute Kidney failure | 584 | D474 , D500 , D501 , D508 , D509 , D510 , D511 , D512 , D513 , D518 , D519 , D520 , D521 , D528 , D529 , D530 , D531 , D532 , D538 , D539 , D550 , D551 , D552 , D553 , D558 , D559 , D560 , D561 , D562 , D563 , D564 , D565 , D568 , D569 , D5700 , D5701 , D5702 , D571 , D5720 , D57211 , D57212 , D57219 , D573 , D5740 , D57411 , D57412 , D57419 , D5780 , D57811 , D57812 , D57819 , D580 , D581 , D582 , D588 , D589 , D590 , D591 , D592 , D593 , D594 , D595 , D596 , D598 , D599 , D600 , D601 , D608 , D609 , D6101 , D6109 , D611 , D612 , D613 , D61810 , D61811 , D61818 , D6182 , D6189 , D619 , D62 , D630 , D631 , D638 , D640 , D641 , D642 , D643 , D644 , D6481 , D6489 , D649 , D65 , D66 , D67 , D680 , D681 , D682 , D68311 , D68312 , D68318 , D6832 , D684 , D6851 , D6852 , D6859 , D6861 , D6862 , D6869 , D688 , D689 , D690 , D691 , D692 , D693 , D6941 , D6942 , D6949 , D6951 , D6959 , D696 , D698 , D699 , D700 , D701 , D702 , D703 , D704 , D708 , D709 , D71 , D720 , D721 , D72810 , D72818 , D72819 , D72820 , D72821 , D72822 , D72823 , D72824 , D72825 , D72828 , D72829 , D7289 , D729 , D730 , D731 , D732 , D733 , D734 , D735 , D7381 , D7389 , D739 , D740 , D748 , D749 , D750 , D751 , D7581 , D7582 , D7589 , D759 , D761 , D762 , D763 , D77 , D892 , I880 , I881 , I888 , I889 , 584 |
| Kidney stone | 590,592,595 | K8000 , K8001 , K8010 , K8011 , K8012 , K8013 , K8018 , K8019 , K8020 , K8021 , K8030 , K8031 , K8032 , K8033 , K8034 , K8035 , K8036 , K8037 , K8040 , K8041 , K8042 , K8043 , K8044 , K8045 , K8046 , K8047 , K8050 , K8051 , K8060 , K8061 , K8062 , K8063 , K8064 , K8065 , K8066 , K8067 , K8070 , K8071 , K8080 , K8081 , K810 , K811 , K812 , K819 , K820 , K821 , K822 , K823 , K824 , K828 , K829 |
| Skin infections | 682,686 | T8141, T8142, L03311 , L03312 , L03313 , L03314 , L03315 , L03316 , L03317 , L03319 , L03321 , L03322 , L03323 , L03324 , L03325 , L03326 , L03327 , L03329 , L03811 , L03818 , L03891 , L03898 , L0390 , L0391 , L080 , L0881 , L0882 , L0889 , L089 , L928 , L983 |
| Altered consciousness | 780 | R400 , R401 , R4020 , R402110 , R402111 , R402112 , R402113 , R402114 , R402120 , R402121 , R402122 , R402123 , R402124 , R402210 , R402211 , R402212 , R402213 , R402214 , R402220 , R402221 , R402222 , R402223 , R402224 , R402310 , R402311 , R402312 , R402313 , R402314 , R402320 , R402321 , R402322 , R402323 , R402324 , R402340 , R402341 , R402342 , R402343 , R402344 , R403 , R404 , R410 , R411 , R412 , R413 , R4182 , R419 , R42 , R440 , R442 , R443 , R531 , R532 , R5381 , R5382 , R5383 , R55 , R6889 |
| Neuromuscular symptoms | 781,9970 | G038 , G970 , G972 , G9731 , G9732 , G9781 , G9782 , I97810 , I97811 , I97820 , I97821 , R250 , R251 , R252 , R253 , R258 , R259 , R260 , R261 , R2681 , R2689 , R269 , R270 , R278 , R279 , R290 , R291 , R293 , R295 , R296 , R29810 , R29818 , R29890 , R29891 , R29898 , R2990 , R2991 , R414 , R430 , R431 , R432 , R438 , R439 , R683 |
| Skin symptoms | 782 | R17 , R200 , R201 , R202 , R203 , R208 , R209 , R21 , R220 , R221 , R222 , R2230 , R2231 , R2232 , R2233 , R2240 , R2241 , R2242 , R2243 , R229 , R230 , R231 , R232 , R233 , R234 , R238 , R239 , R600 , R601 , R609 |
| Nutrition symptoms |  | R627 , R630 , R631 , R632 , R633 , R634 , R635 , R636 , R638 |
| Cardiovascular symptoms | 785,9971,9972,9977 | I96 , I97110 , I97111 , I97120 , I97121 , I97130 , I97131 , I97190 , I97191 , I97710 , I97711 , I97790 , I97791 , I9788 , I9789 , R000 , R002 , R008 , R009 , R011 , R012 , R0989 , R570 , R571 , R578 , R579 , R6521 , T81710A , T81711A , T81718A , T81719A , T8172XA |
| Respiratory symptoms |  | J954 , J955 , J95859 , J9588 , J9589 , R042 , R0481 , R0489 , R049 , R05 , R0600 , R0601 , R0602 , R0609 , R061 , R062 , R063 , R064 , R066 , R0681 , R0682 , R0683 , R0689 , R069 , R071 , R072 , R0781 , R0782 , R0789 , R079 , R093 , R0989 , R222 |
| Heartburn | 7871 | R12 |
| Dysphagia | 7872 | R130 , R131, R1310 , R1311 , R1312 , R1313 , R1314 , R1319 |
| Gas | 7873 | R140 , R141 , R142 , R143 |
| Other digestive symptoms | 7874,7875,7876,7877,7879,9974 | K5229 , K5289 , K9130 , K9131 , K9132 , K9181 , K9182 , K9183 , K9186 , K9189 , R150 , R151 , R152 , R159 , R1911 , R1912 , R1915 , R192 , R194 , R195 , R197 , R198 |
| Urinary symptoms |  | N23 , N393 , N3941 , N3942 , N3943 , N3944 , N3945 , N3946 , N39490 , N39491 , N39492 , N39498 , N990 , N99520 , N99521 , N99522 , N99523 , N99524 , N99528 , N99530 , N99531 , N99532 , N99533 , N99534 , N99538 , N9981 , N9989 , R300 , R301 , R309 , R32 , R330 , R338 , R339 , R34 , R350 , R351 , R358 , R360 , R369 , R390 , R3911 , R3912 , R3913 , R3914 , R3915 , R3916 , R39191 , R39192 , R39198 , R392 , R3981 , R3982 , R3989 , R399 |
| Sudden death | 798 | R99 |
| Foreign body in mouth, esophagus, and stomach | 935 | T180XXA , T18100A , T18108A , T18110A , T18118A , T18120A , T18128A , T18190A , T18198A , T182XXA |
| Sepsis | 9959 | A021 , A227 , A267 , A327 , A400 , A401 , A403 , A408 , A409 , A4101 , A4102 , A411 , A412 , A413 , A414 , A4150 , A4151 , A4152 , A4153 , A4159 , A4181 , A4189 , A419 , A427 , A5486 , B377 , R6510 , R6511 , R6520 , R6521 |
| Hemorrhage | 99811,99812 | D7801 , D7802 , D7821 , D7822 , D7831 , D7832 , E3601 , E3602 , E89810 , E89811 , E89820 , E89821 , G9731 , G9732 , G9751 , G9752 , G9761 , G9762 , I97410 , I97411 , I97418 , I9742 , I97610 , I97611 , I97618 , I97620 , I97621 , I97630 , I97631 , I97638 , J9561 , J9562 , J95830 , J95831 , J95860 , J95861 , K9161 , K9162 , K91840 , K91841 , K91870 , K91871 , L7601 , L7602 , L7621 , L7622 , L7631 , L7632 , N9961 , N9962 , N99820 , N99821 , N99840 , N99841 |
| Disruption of internal operation (surgical) wound | 99831 | T8132XA |
| Wound adhesions | 99832,9985 | K6811, T8131XA, T814XXA |
| Abnormal reaction | E878,E879 | Y831 , Y832 , Y838 , Y839 , Y840 , Y841 , Y842 , Y843 , Y844 , Y845 , Y846 , Y847 , Y848 , Y849 |
| Sudden arrest | V1253 | Z8674 |
| Poor diet | V691 | Z724 |

**Revisional CPT codes:**

**Conversions**

Unlisted stomach procedure 43999

Excision, local; ulcer of stomach 43610

Other procedures on the stomach 43840

Unlisted laparoscopy procedure, stomach 43659

Conversion to RYGB (CPT 43644, 43645, 43846, and 43847)

**Revisions**

Band revision or removal 43771, 43772, 43773, 43774, 43886, 43887, 43888 43775 Revision sleeve gastrectomy 43775

Revision gastroplasty, Revision, open, of gastric restrictive procedure for morbid obesity, other than adjustable gastric restrictive device. 43848

Revision of gastroduodenal anastomosis (gastroduodenostomy) with reconstruction; without vagotomy 43850

Revision of gastroduodenal anastomosis (gastroduodenostomy) with reconstruction; with vagotomy 43855

Revision of gastrojejunal anastomosis (gastrojejunostomy) with reconstruction with or without partial gastrectomy or intestine resection; without vagotomy 43860

Revision of gastrojejunal anastomosis (gastrojejunostomy) with reconstruction, with or without partial gastrectomy or intestine resection; with vagotomy 43865

Revision of gastrojejunal anastomosis (gastrojejunostomy) with reconstruction, with or without partial gastrectomy or intestine resection; without vagotomy 43869

Enterectomy 44120

Laparoscopic Enterectomy 44202 and 44203

Unlisted lap procedure on intestine 44238

Volvulus/internal hernia 44050

Lap enterolysis 44180

Suture of mesentery 44850

Lap unlisted 49329

Cholecystectomy 47562 and 47563

**Esophageal procedures**

43235 – EGD with brushing/washing.

43236 – EGD with directed injection.

43239 – EGD with biopsy.

43247 – removal foreign body.

43249 – balloon dilation <30mm.

43233 – balloon dilation 30mm or more.

43245 – dilation of gastric/duodenal strictures

control of bleeding 43255

stent placement 43266

**Surgical interventions CPT codes:**

General unlisted laparoscopic procedures on the intestine or stomach (44238, 43999, 43659)

Postoperative perforation, leak, and ulcer related (43610, 43840, 49905, 49329)

Internal hernia or bowel obstruction

(44050, 44180, 44850)
